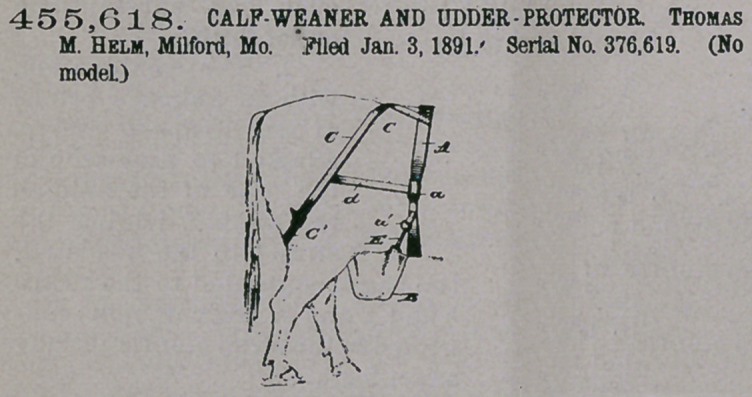# Recent Patents

**Published:** 1891-08

**Authors:** 


### RECENT PATENTS

RELATING TO

### VETERINARY MEDICINE AND ANIMAL INDUSTRY.

*Issued by U. S. Patent Office for Month ending July,* 1891.

**454,851.** HORSESHOE. GEORGE T. CHAPMAN, White Plains, N. Y., assignor of one-half to William Harvey Merritt, New York, N. Y. Filed Aug. 8, 1889. Serial No. 320,169. (No model.)

*Claim.*—1. In a heel-expanding shoe consisting of two parts divided longitudinally and unconnected at the toe, said parts each having a joint-lug on the inner edge about midway between the toe and heel, and separately pivoted thereby to the respective extremeties of the connecting-bar, coupling the two parts at pivotal points located at the inner edges, or thereabout, of the said parts, respectively, substantially as described.

2. The improved heel-expanding horseshoe, consisting of two parts divided longitudinally at the toe, each part having a joint-lug on the inner edge about midway between the toe and the heel, and said parts connected together by the cross-bar pivoted at the ends to the joint-lugs of the respective parts of the shoe, and by an expanding-screw at one end, substantially as described.

3. The improved expanding horseshoe, consisting of two parts divided longitudinally and unconnected at the toe, each part having the joint-lug on the inner edge about midway between the toe and the heel, and said parts connected together by the cross-bar pivoted at the ends to the joint-lugs of the respective parts of the shoe, and by the heel-expanding screw, substantially as described.

4. The improved heel-expanding horseshoe, consisting of two parts divided longitudinally at the toe and having the heel-expanding clips, and being joined together between the toe and the heel by the bar pivoted at the ends to the joint-lugs of the respective parts and coupled at one end by an expanding-screw, substantially as described.

5. The improved heel-expanding horseshoe, consisting of two parts divided longitudinally at the toe, joined together between the toe and heel by the bar and joint-lugs, and having the heel-expanding rod fitted in eye-terminals of the heel extended upward above the level of the shoe, substantially as described.

6. The improved heel-expanding horseshoe, consisting of two parts divided longitudinally at the toe, jointed together between the toe and heel by the bar and joint-lugs, and having the heel-expanding rod and toe-clips, all substantially as described.

7. The improved heel-expanding horseshoe, consisting of two parts divided longitudinally at the toe, joined together between the toe and the heel by the bar and joint-lugs, and having the heel-expanding clips, substantially as described.

**454,501. ANIMAL-STOCK. William Stemem, Bremen, Ohio.** Filed Feb. 9, 1891. Serial No. 380,799. (No model.)

*Claim.*—1. In an animal-stock, a base, uprights carried thereby, and cross-pieces secured to the uprights, in combination with sliding jaws mounted between the cross-pieces, and levers carried by the uprights and connecting with the jaws for opening and closing the same.

2. In an animal-stock, a base, uprights carried thereby, and cross-pieces secured to the uprights, in combination with sliding jaws mounted between the cross-pieces, mecanism for opening and closing the jaws, and adjustable side wings hinged adjacent to and adapted to move laterally in unison with the said jaws.

3. In an animal-stock, a base, uprights carried thereby, and cross-pieces secured to the uprights, in combination with rods carried by the crosspieces, side wings having a sliding connection with the rods, and mechanism for raising or lowering the said wings.

4. In an animal-stock, a base, uprights carried thereby, a cross-piece secured to the upper ends of the uprights, and slotted cross-pieces secured to the uprights between the base and the top cross-piece, in combination with rods carried by the slotted cross-pieces, side wings having collars loosely engaging the rods, collars located between the collars of said wings and having lugs thereon, a lever carried by the top cross-piece, the mechanism connecting the lever and the lugs, whereby when the lever is actuated the side wings will be correspondingly raised or lowered.

5. In an animal stock, the combination, with movable side wings, of a breeching-strap for securing the wings together and spring-actuated back-straps carried by one of the wings and having perforations designed to engage projections on the other wing.

6. In an animal-stock, the combination, with movable side wings, of spring-actuated back-straps carried by one of the wings and having perforations designed to engage projections on the other wing.

7. An animal-stock having vertically and laterally adjustable side wings, in combination with a ratchet and-pawl mechanism and a series of pulleys carried by each wing and a rope designed to engage each series of pulleys, one end of each of the ropes being secured to the ratchet mechanism and the other end being provided with a clasp.

**454,885. LEG-SPREADER FOR HORSES. WILLIAM SMITH and THEOPHILUS E. HILLS, Heron Lake, Minn. Filed Sept. 9, 1890. Serial No. 364,439. (No model.)**

*Claim.*—1. In a device of the character described, the combination, with the shafts of the vehicle, and cords extending from said arms beyond the sides of the shaft downward and adapted to be connected to the adjacent hind foot of a horse, substantially as and for the purposes set forth.

2. A leg-spreader comprising in combination a rigid outwardly-projecting arm arranged upon the vehicle-shaft, an exterior guard upon said shaft extending outside of said arm, and a flexible elastic cord secured to said arm and adapted to be detachably connected to the hind foot of the horse between said shafts, substantially as and for the purposes set forth.

3. In a device of the class described, the combination of the rigid outwardly-projecting arm 3, adjustable lengthwise of the shaft of the vehicle, the strap 14, adapted to be secured between the hoof and its shoe and projecting therefrom, and the elastic cord 8, adapted to connect said arm with said strap, substantially as and for the purposes set forth.

4. In a device of the class described, the combination, with the vehicle, of sheaves having rigid support upon the vehicle-shaft above and outside the hind feet of the horse, and a cord running over said sheaves on opposite sides and to the rear of the horse and adapted to have its ends respectively detachably connected to the hind feet of the horse, substantially as and for the purposes set forth.

**454,473. VETERINARY INCISOR-CUTTER. HERMANN HAUSSMANN, Chicago, Ill. Filed June 25, 1889. Serial No. 315,553. (No model.)**

*Claim.*—1. In a veterinary incisor-cutter, the combination, with the cutting-jaw, of a sustaining-jaw and a pivot connecting said jaws forward of the sustaining-jaw being located to the rear of the cutting-jaw and to which access is gained between the cutting-jaw and the pivot, whereby the tooth is inserted between the cutting-jaw and the pivot and the outer face thereof is supported by the sustaing-jaw, substantially as described.

2. In a veterinary incisor-cutter, the combination, with the handle B, provided at its forward end with a cutting-jaw, and the lugs D, terminating approximately in a plane parallel with the cutting-jaw and perpendicular to the handle, of the handle A, pivoted at its forward end between said lugs, and a sustaing-jaw, substantially as described.

**455,472.** BRIDLE. LOUIS DANNHAUSER, Munich, Germany. Filed Apr. 7, 1891. Serial No. 388,036. (No model.)

*Claim.*—1. The combination, with an animal's bridle and ordinary reigns, of a strap adapted to pass around the nose of the animal, a bag of air-tight material connected thereto and adapted to be extended or pulled over the nostrils of the animal, and an additional rein attached to the free end of such bag, substantially as set forth.

2. The combination, with an animal's bridle, of the straps *b* and *g*, adapted to pass around the nose of the animal, the strap *f*, joining the strap *b* to the headstall, the bag *h*, connected to the straps *b* and *g*, and a reign or reins connected to the strap *g*, substantially as set forth.

3. The combination, with an animal's bridle, of the straps *b* and *g*, adapted to pass around the nose of the animal, the strap *f*, joining the strap *b* to the headstall, the bag *h*, connected to straps *b* and *g*, the rein *c*, connected to the strap *g*, and the guide-ring *c*1, substantially as set forth.

**455,690.** SUPPLEMENTAL HORSESHOE. HERBERT S. PULLMAN, Meriden, Conn., assignor of one-half to S. W. Kent, same place. Filed Jan. 12, 1891. Serial No. 377,464. (No model.)

*Claim.*—1. The combination of the toe-piece adapted to engage the shoe at the toe end, two arms E E, hinged thereto, extending rearward, their rear ends adapted to engage the shoe at the rear upon its outsides, levers H H, hung, respectively, to the arms E E, near their rear ends upon the inside of the shoe, one arm of said levers adapted to engage the inside of the shoe, as opposed to the engagement of the arms upon the outside, a cross-head hung to the other arms of the said levers, and a bolt through said cross-head connected with said toe-piece, whereby the said cross-head may be forced toward the toe-piece and correspondingly turn the levers, supstantially as described.

2. The combination of the toe-piece B, adapted to engage the shoe at the toe, the two arms E E, hung to said toe-piece and extending rearward, their real ends turned upward, so that each may engage the shoe upon the outer opposite sides of the shoe, L-shaped levers H H, hung to the said arms E E upon the inside of the shoe, one arm of said levers adapted to engage the shoe upon the insides and opposed to the engagement of the arms upon the outsides, the said ends of the levers conrtructed with flanges I to pass in over the top of the shoe, a cross-head J, hung to the other arms of said levers, with a bolt L through said cross-head and connected with the toe-piece, substantially as described.

3. The combination of a toe-piece B, adapted to engage the shoe at the toe, two arms E E, hung to the said toe-piece and extending rearward, their rear ends upturned to engage the shoe upon the outsides at the rear, levers H H, hung, respectively, to the arms E E near their rear ends, one arm of said levers adapted to engage the shoe upon the insides, opposed to the engagement of the arms upon the outsides of the shoe, a cross-head J, hung to the other arms of the said levers, with a bolt through said cross-head and connected with the toe-piece, and the hole through the said cross-head through which the bolt passes, enlarged from the sutside inward to permit the rocking movement of the cross-head on the bolt, substantially as described.

455,618. **CALF-WEANER AND UDDER-PROTECTOR.** THOMAS M. HELM, Milford, Mo. Filed Jan. 3, 1891. Serial No. 376,619. (No model.)

*Claim.*—1. In combination with a pouch B, having attached to one side thereof a snap-hook E and adjacent to the opposite side of the pouch a strap A and straps C1 C1, the strap A having a ring with which the snap-hook engages, and straps C C attached to the strap A and adapted to engage with the straps C1 C1, substantially as shown, and for the purpose set forth.

2. In an udder-protector, the combination of the flexible connections A, C, and C1, connected to each other substantially as shown, straps *d*, connecting the straps A and C C with each other above their points of adjustment, a pouch secured to the strap A, said pouch also having straps C1 and a snap-hook on its longer side for engagement with the ring *a*1, and buckles, substantially as set forth.

## Figures and Tables

**Figure f1:**
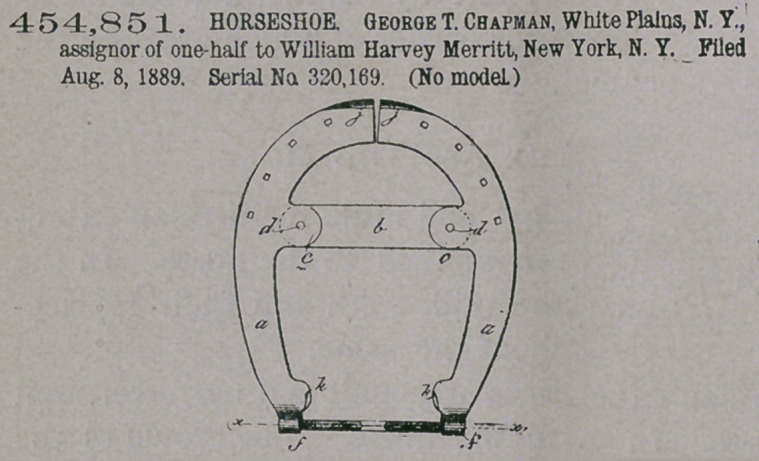


**Figure f2:**
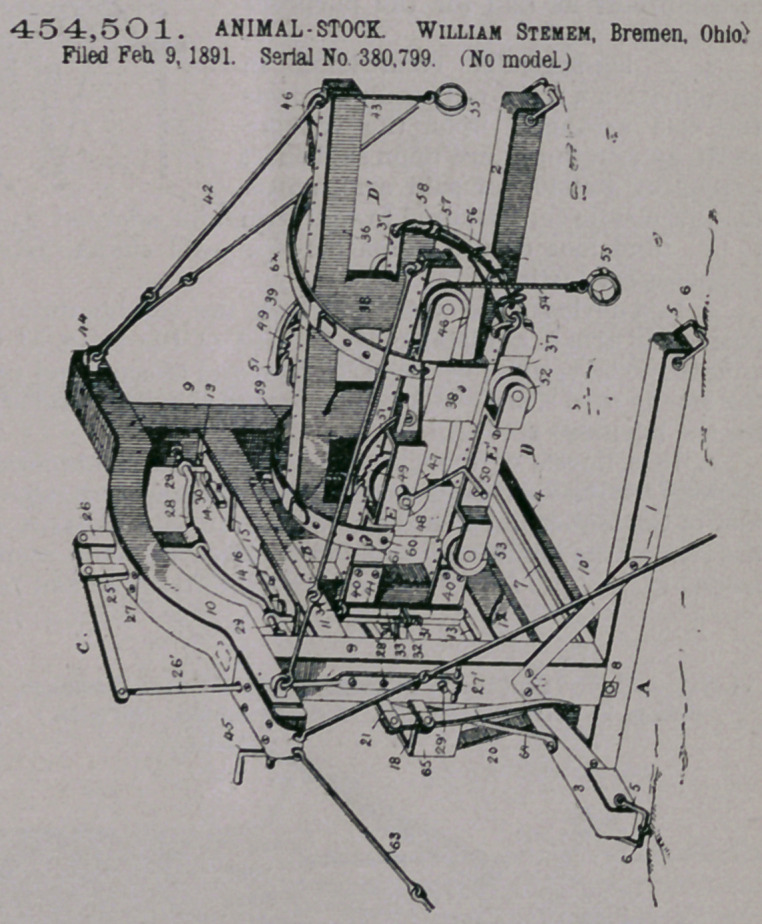


**Figure f3:**
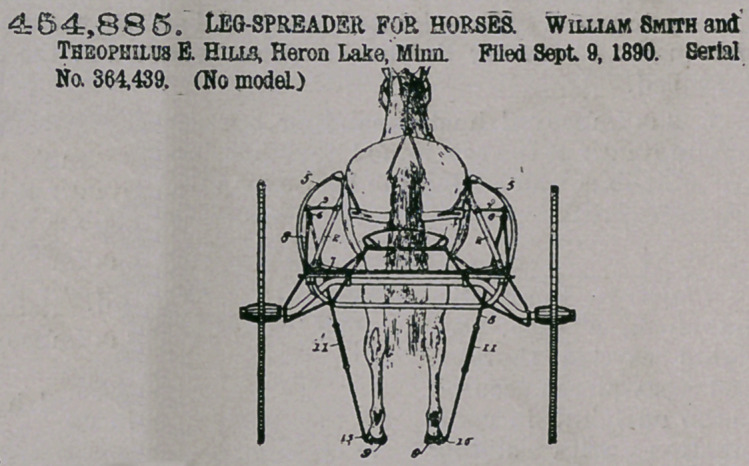


**Figure f4:**
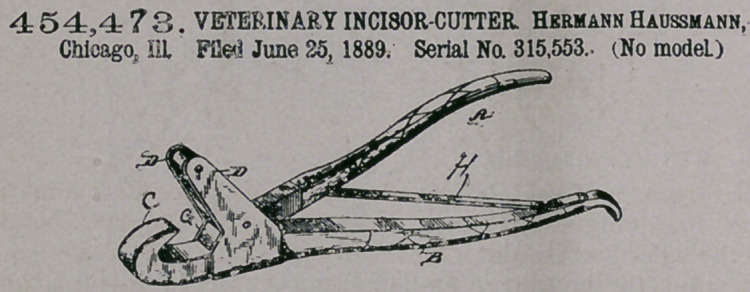


**Figure f5:**
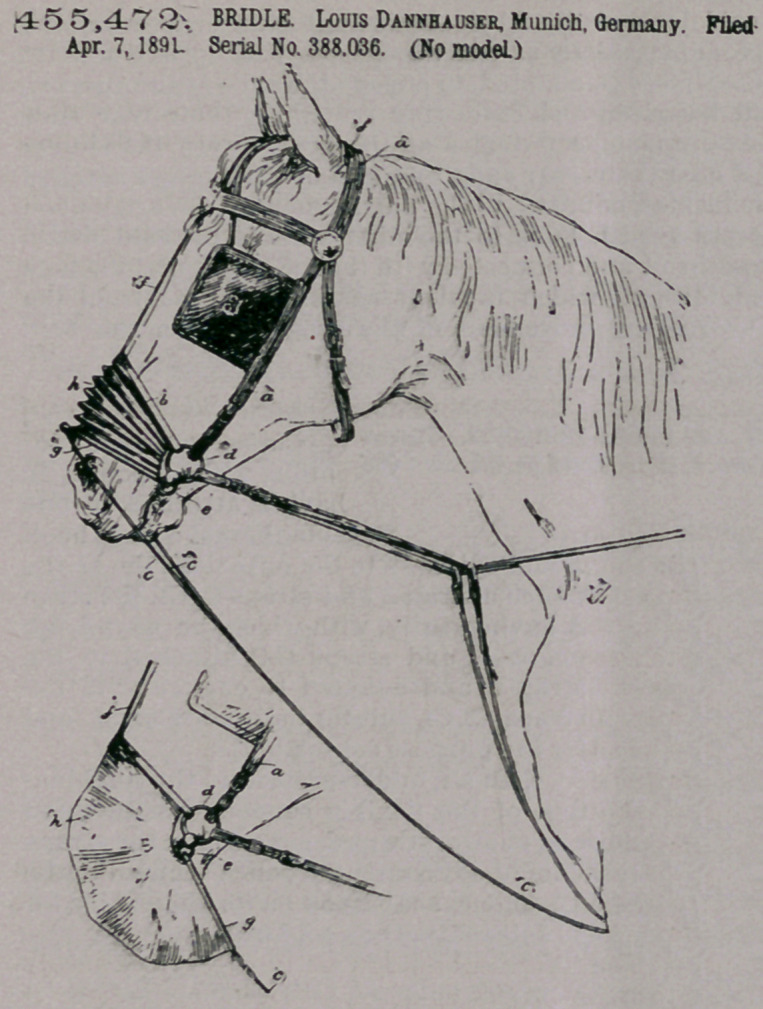


**Figure f6:**
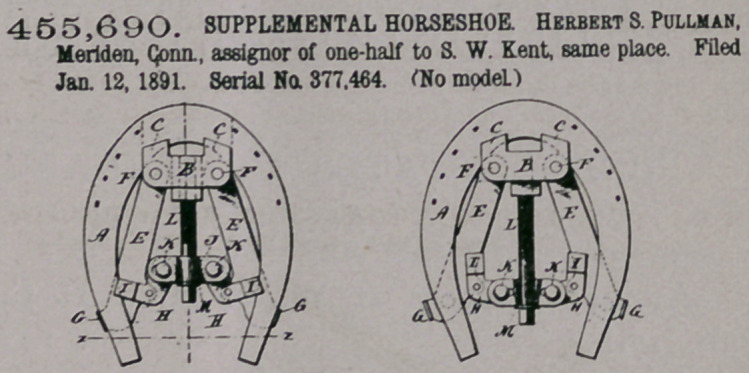


**Figure f7:**